# Cysteine Disulfides (Cys-ss-X) as Sensitive Plasma Biomarkers of Oxidative Stress

**DOI:** 10.1038/s41598-018-35566-2

**Published:** 2019-01-14

**Authors:** Xiaoyun Fu, Shelby A. Cate, Melissa Dominguez, Warren Osborn, Tahsin Özpolat, Barbara A. Konkle, Junmei Chen, José A. López

**Affiliations:** 1 Bloodworks Research Institute, Seattle, Washington USA; 2 Department of Medicine, University of Washington, Seattle, Washington USA

## Abstract

We developed a high-throughput mass spectrometry–based method to simultaneously quantify numerous small-molecule thiols and disulfides in blood plasma. Application of this assay to analyze plasma from patients with known oxidative stress (sickle cell disease and sepsis) and from a patient with sickle cell disease treated with the antioxidant N-acetylcysteine suggests that cysteine disulfides, in particular protein-bound cysteine, serve as sensitive plasma biomarkers for the extent of oxidative stress and effectiveness of antioxidant treatment.

## Introduction

Oxidative stress accompanies a wide variety of diseases^1^, including sickle cell disease (SCD), HIV/AIDS, and rheumatoid arthritis, and antioxidant therapy is emerging as a pharmacological strategy for treating diseases in which oxidative stress is known or suspected to be elevated^2^. The ability to measure oxidative stress quantitatively is important for understanding disease mechanisms and monitoring the effectiveness of antioxidant treatments. Among biomarkers of oxidative stress, the ratio of reduced glutathione (GSH) to glutathione disulfide (GSSG) is frequently measured in various cell types, owing to the millimolar intracellular concentrations of these glutathione species and the broad availability of assays for their measurement, including many that are commercially available^1,3,4^. Despite these advantages, GSH/GSSG is not well suited as a plasma biomarker of oxidative stress due to the low plasma concentrations of GSH species, which are usually in the low micromolar range, and the low sensitivity of the assays. A potentially better biomarker for oxidative stress in plasma is the amino acid cysteine (Cys) and its oxidized forms, including cystine and mixed disulfides with other thiol-containing small molecules and proteins. In plasma, the concentration of Cys species is more than 20 times higher than that of GSH species^5^. Various methods have been described for measuring thiols and disulfides in plasma, but because of the dynamic nature of thio-disulfide exchange reactions, these measurements have remained challenging^6^.

To test the feasibility of using Cys and its oxidized species as a measure of oxidative stress, we developed a high-throughput quantitative assay for the simultaneous analysis of a panel of small-molecule thiols and disulfides by ultra-performance liquid chromatography-tandem mass spectrometry (UPLC-MS/MS) using multiple reaction monitoring (MRM) coupled with stable isotope dilution. After optimizing sample acquisition, processing, and analysis, we applied this method to characterize Cys species in plasma from normal donors and patients with different diseases, and to monitor changes in plasma Cys redox state in an SCD patient being treated with N-acetylcysteine (NAC), an FDA-approved antioxidant drug.

**Figure 1:**
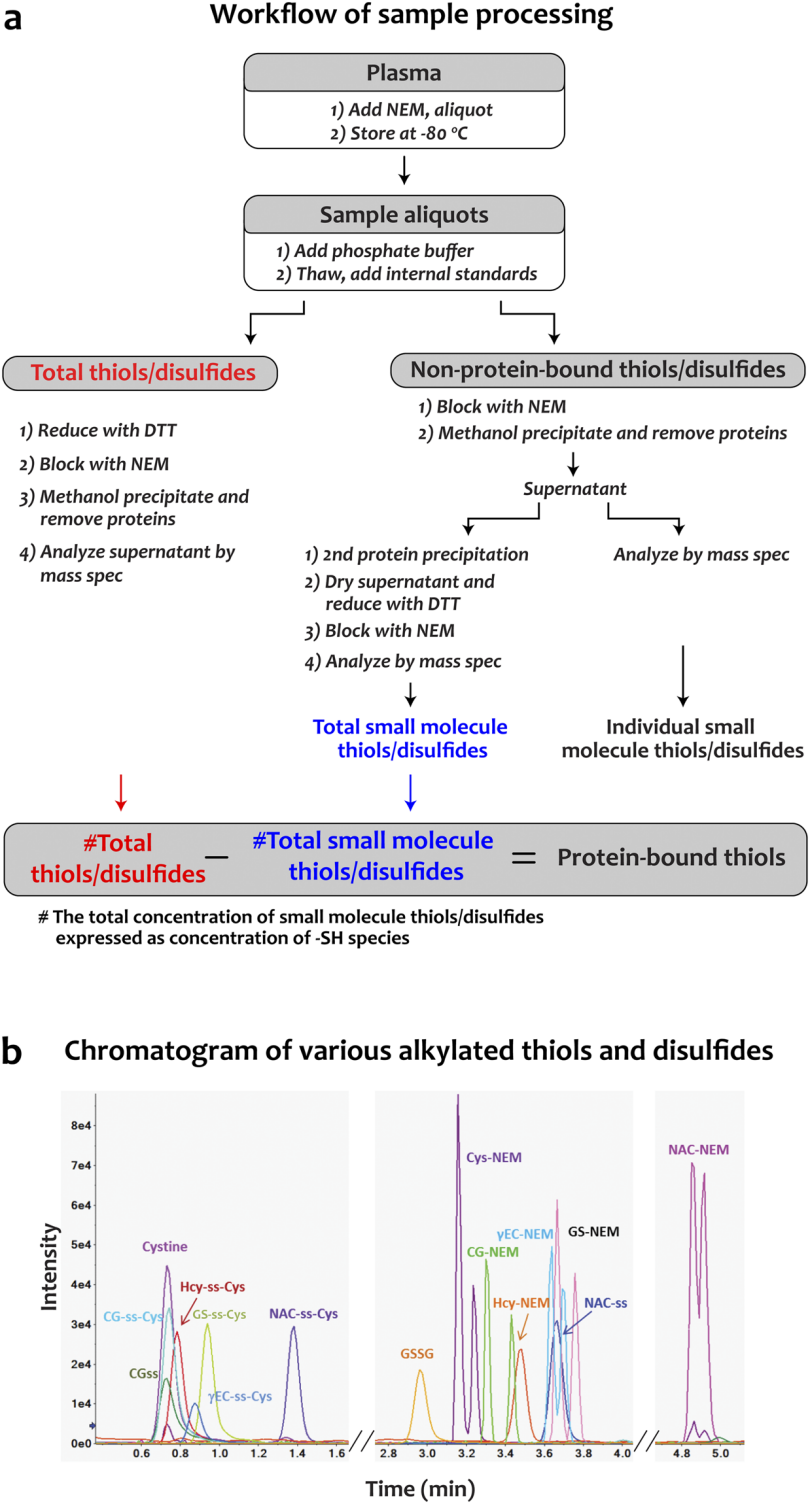
Quantification of glutathione and cysteine species by LC-MS/MS-MRM. (**a**) Workflow for sample processing for MS analysis. (**b**) A representative UPLC chromatogram for a panel of disulfides and NEM-alkylated thiols.

One of the biggest challenges in analyzing thiols and disulfides is avoiding artefactual oxidation^7^ and thiol-disulfide exchange^6^ during sample preparation and analysis. We addressed these problems by first blocking thiols with N-ethylmaleimide (NEM), which minimizes both problems^8^. Figure 1a shows workflows to quantify individual small molecule thiols and disulfides, total small molecule thiols (reduced and disulfide forms), and total thiols (including protein-bound forms) in biological samples as described in the Methods. Isotopically labeled internal standards were used for absolute quantification. Proteins in the samples were precipitated with methanol and the supernatants were analyzed. The disulfides and thiol-NEM adducts were separated by UPLC and detected by LC-MS/MS-MRM. Product ions from the analyte were quantified by comparison to analogous ions derived from isotopically labeled internal standards (equation 1, Methods). We optimized MRM conditions to ensure maximum signal and sensitivity for each analyte. Under optimized conditions, all analytes eluted from the LC column within 5 minutes (Fig. 1b). This quantification method offered good intra- and inter-day reproducibility, wide linear dynamic ranges, and high sensitivity for analyte detection in complex biological samples. (Supplementary Table 1).

**Figure 2:**
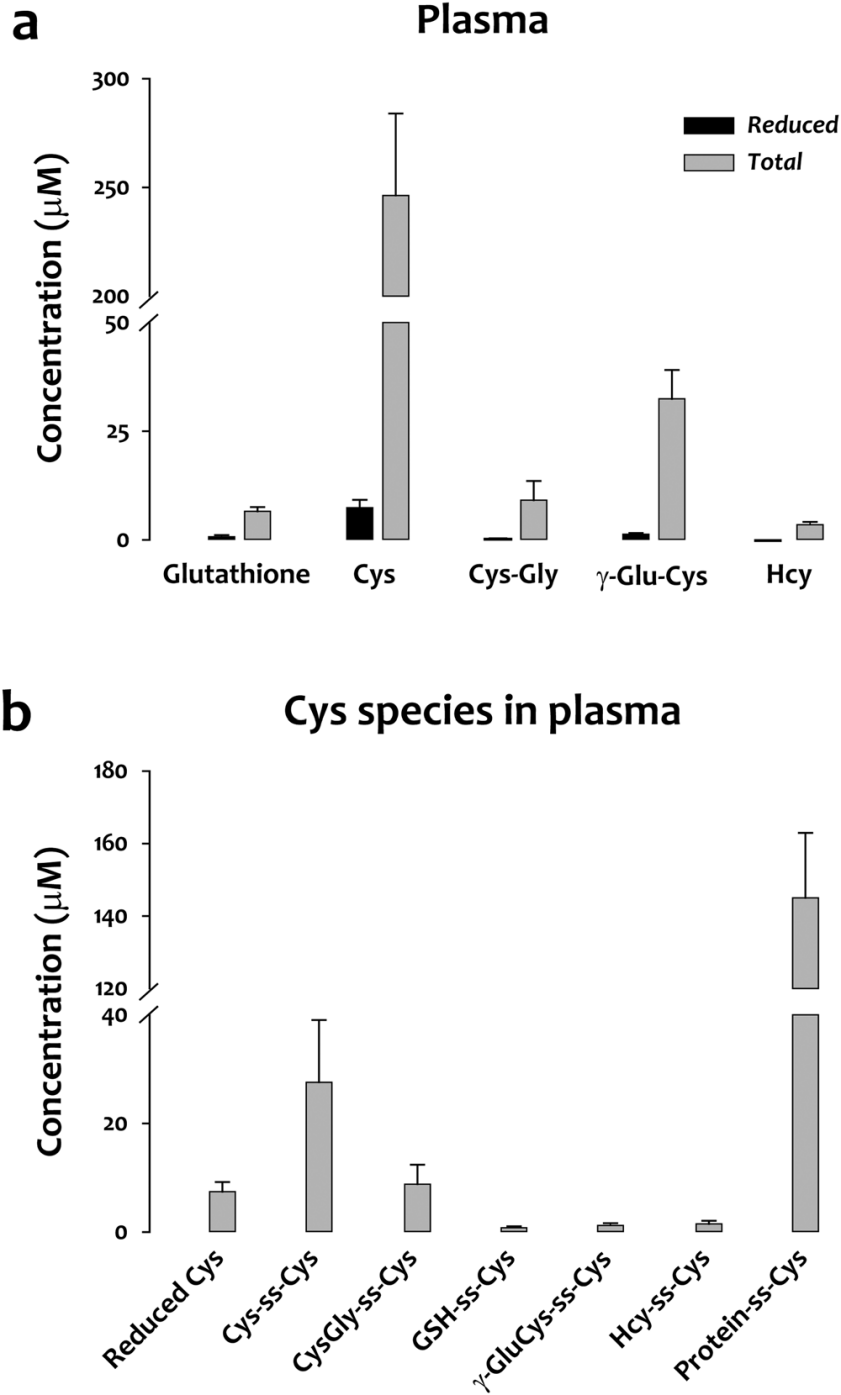
Cysteine, not glutathione, is the major thiol-containing compound in plasma. (**a**) Plasma concentrations of reduced and total forms of glutathione, cysteine (Cys), Cys-Gly, γ-Glu-Cys, and homocysteine (Hcy). (**b**) Concentrations of Cys either in reduced form or disulfide-bonded adducts measured in plasma. Data in panels (**a**) and (**b**) are from 11 healthy donors.

We applied this assay to analyze whole blood and plasma samples from 11 heathy donors. In whole blood, reduced glutathione was the most abundant thiol, with a concentration of approximately 1 mM (Supplementary Fig. 1). In plasma, Cys species were most abundant, with a total concentration of approximately 250 µM, less than 2% existing in the reduced form (Fig. 2a). We also quantified the major Cys-containing disulfides in plasma, including cystine (Cys-ss-Cys), mixed disulfides (GSH-ss-Cys, CysGly-ss-Cys, γGluCys-ss-Cys, and Hcy-ss-Cys), and protein-bound Cys (protein-ss-Cys) (Fig. 2b). Interestingly, protein-bound Cys was the most abundant form of Cys in plasma (145 ± 18 μM). Protein-bound glutathione has long been considered an indicator of oxidative stress in whole blood^1^, but much less attention has been paid to protein-bound Cys. One reason that protein-bound Cys has been overlooked is the lack of a sensitive, quantitative, and high-throughput assay. Here, we determined the protein-bound Cys concentration by subtracting the concentration of total small molecule Cys from that of total Cys as shown in Fig. 1a.

**Figure 3:**
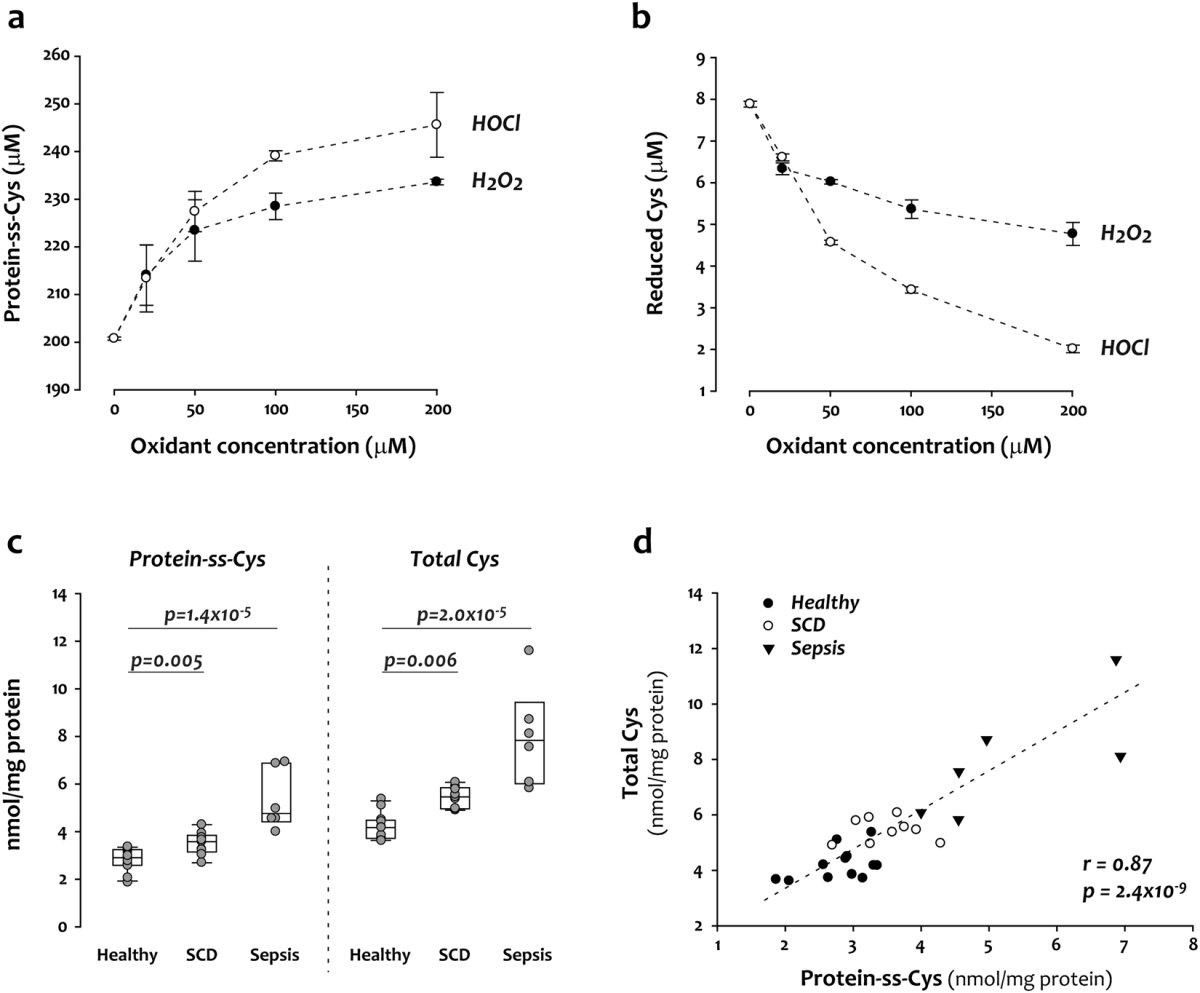
Protein-bound cysteine (protein-ss-Cys) in plasma is a biomarker for oxidative stress. (**a**,**b**) Oxidization of plasma by H_2_O_2_ and HOCl. The concentrations of protein-ss-Cys increased in plasma as oxidant concentration increased (**a**) while reduced Cys decreased (**b**). (**c**) Compared to healthy donors (n = 12), patients with SCD (n = 9), or sepsis (n = 6) had significantly elevated protein-ss-Cys and total Cys concentrations in plasma. (**d**) The concentration of protein-ss-Cys correlated positively with the total Cys concentration in plasma. For sepsis, the data are derived from 6 samples from 5 patients and the statistics are calculated based on n = 6. The 2 samples from one patient (patient 3 in Supplementary Table 3) were taken 3 days apart.

To understand how oxidation in plasma affects the concentration of protein-bound Cys, we incubated plasma with hydrogen peroxide (H_2_O_2_) or the neutrophil-derived oxidant hypochlorous acid (HOCl), two oxidants generated *in vivo*. Both oxidants increased the concentration of protein-bound Cys and decreased the concentration of reduced Cys in a concentration-dependent manner (Fig. 3a,b). We next measured total Cys and protein-bound Cys in plasma from 2 sets of patients with conditions associated with oxidative stress, SCD and sepsis (see clinical information in Supplementary Tables 2,3). Both total Cys and protein-bound Cys were significantly elevated in the two groups of patients as compared to healthy donors (Fig. 3c). The increase in total Cys correlated linearly with increased protein-bound Cys (Fig. 3d).

**Figure 4:**
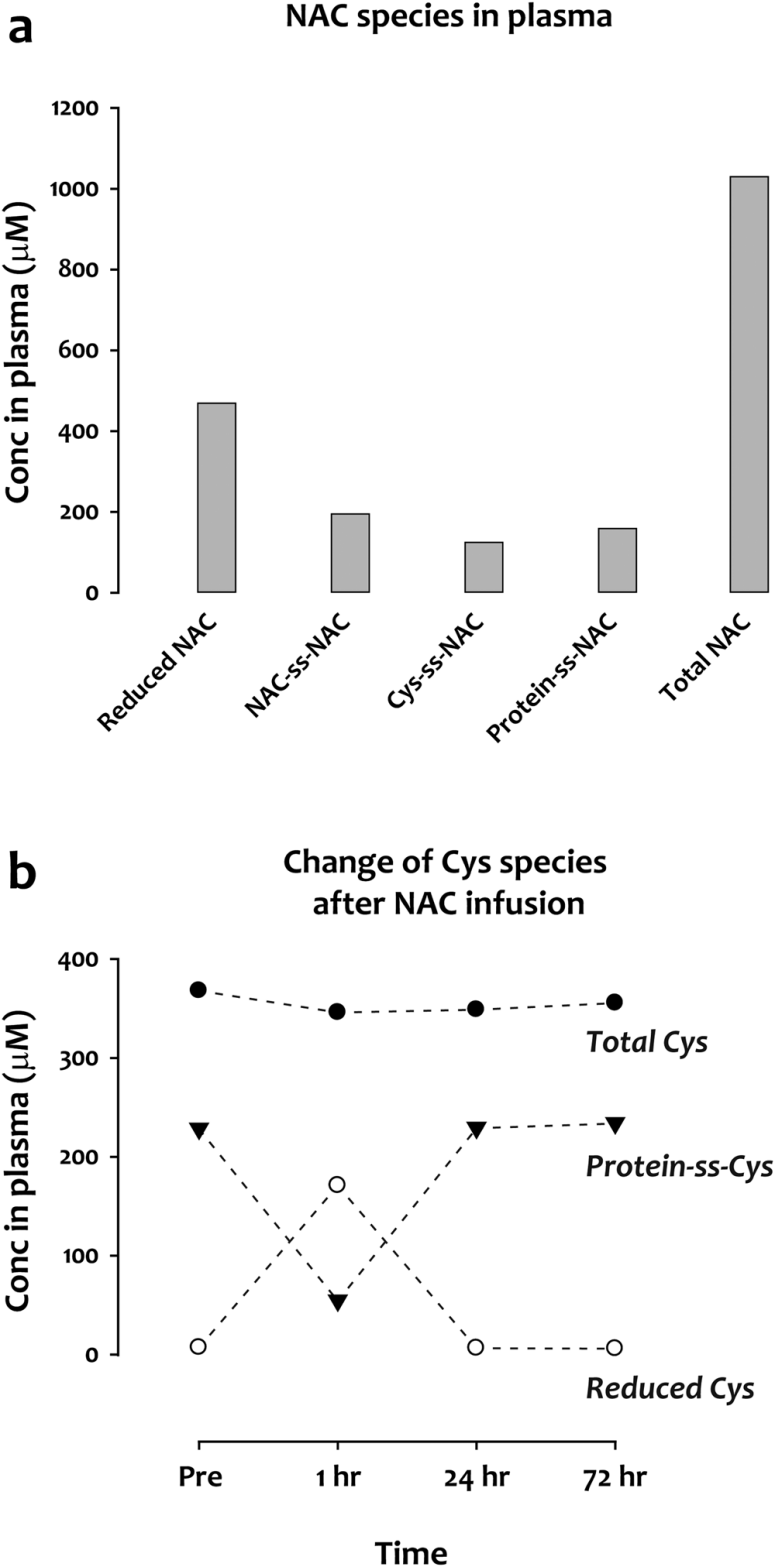
Changes of plasma Cys species in an SCD patient received NAC infusion. An SCD patient was infused with NAC, 150 mg/kg over 1 hour. (**a**) Concentrations of NAC and its disulfide-bonded forms measured at the end of the infusion. (**b**) Time course of concentrations of total, reduced, and protein-bound Cys before and after NAC infusion.

NAC has been used for over half century to treat acetaminophen overdose and chronic obstructive lung disease^9^. To determine how NAC affects plasma thiol/disulfide concentrations, we analyzed plasma samples from an SCD patient who received 150 mg/kg of NAC intravenously infused over 1 hr. The blood was sampled at baseline (pre), at 1 hr (immediately after the infusion), and at 24 and 72 hr after the start of the infusion. Despite the fact that 99.5% of the NAC was in the reduced form prior to infusion (data not shown), 55% of the NAC present in plasma at 1 hr was oxidized, forming homo- and mixed disulfides. The total concentration of NAC-containing species reached about 1 mM at 1 hr (Fig. 4a). Although there were no significant changes for total Cys upon NAC treatment, reduced Cys increased dramatically, from 7 μM at baseline to 171 μM at 1 hr, accompanied by an almost identical decrease in protein-bound Cys (Fig. 4b). These results suggest that, upon entering the blood, NAC reduces disulfide bonds and releases the reduced form of Cys, primarily from proteins. This mechanism is at odds with the previously proposed mechanism in which Cys is derived from NAC primarily by enzymatic deacetylation^10,11^ and/or reduction of cystine^12,13^. Our analysis provides an instructive example of the usefulness of the assay for mechanistic studies.

In summary, we have developed a sensitive high-throughput method to simultaneously quantify a panel of thiols and disulfides in plasma. In this assay, we use NEM to protect sensitive thiols from oxidation during sample preparation, add isotopically labeled internal standards for absolute quantification, and detect specific ions of each analyte by MRM to minimize the background from plasma. Furthermore, we showed that plasma protein-bound Cys is a sensitive biomarker of oxidative stress: its concentration was elevated in patients under oxidative stress and decreased by treatment with the antioxidant NAC. In addition, this method allowed us to elucidate the mechanism by which NAC generates Cys in plasma, a mechanism that is different from the one proposed by prevailing theory. To our knowledge, this is the most comprehensive method available to analyze thiols and disulfides in plasma. This method is useful for monitoring patients for oxidative stress and evaluating the effectiveness of antioxidant treatments.

## Methods

### Patients and healthy donors

Blood collection from healthy donors was approved by the Western Institutional Review Board, and blood collection from patients with sepsis or SCD at disease baseline with or without NAC infusion was approved by the Institutional Review Board of the University of Washington. The SCD patient who received NAC infusion was enrolled in a pilot clinical trial (NCT01800526) to examine the safety of NAC infusion in patients with SCD. A total of 18 healthy donors were used. The experiments shown in Fig. 2 and Supplementary Fig. 1 involve 11 healthy donors; the experiments shown in Fig. 3c,d involve 12 healthy donors, 5 of whom are also used in the experiments in Fig. 2. Written informed consents were obtained from all study participants and all experiments performed in this study complied with the IRB guidelines and regulations.

### Blood sample collection

Blood was drawn into 3.2% sodium citrate vacutainers. Blood was pooled from all the vacutainers and total volumes of the collected blood and sodium citrate were recorded to calculate the dilution factor. To prepare plasma, blood was subjected to two sequential centrifugations in a centrifuge with swinging buckets. The first spin was at 120 g for 15 min at room temperature (RT) with the brake set at low. The top layer, containing platelet-rich plasma, was collected and centrifuged again at 1,200 g for 10 min at RT, platelet-poor plasma was collected from the supernatant and the platelet pellets were discarded. N-ethylmaleimide (NEM, Pierce) dissolved in phosphate-buffered saline (PBS) was added to plasma to a final concentration of 5 mM. Plasma samples were then aliquoted, snap-frozen in liquid nitrogen, and stored at −80 °C until analysis.

**Table 1:** MRM transitions and optimized conditions.

Transition name	Precursor (*m/z*)	Product (*m/z*)	DP (V)	CE (V)
Cystine-74	241.0	74.0	30	40
Cystine*-77	249.1	77.0	30	40
Cystine-120	241.0	120.0	30	25
Cystine*-124	249.1	124.0	30	25
CG-ss-177	355.1	177.0	40	27
*CG-ss-235*	355.1	235.1	40	25
CG-ss-Cys-177	298.1	177.0	40	25
*CG-ss-Cys-130*	298.1	130.0	40	25
*γEC-ss-Cys-241*	370.1	241.0	40	25
*γEC-ss-Cys-152*	370.1	152.0	40	30
Hcy-ss-Cys-88	255.1	88.0	20	40
Hcy-ss-Cys-134	255.1	134.0	20	20
GSH-ss-Cys-298	427.1	298.0	30	20
GSH-ss-Cys-231	427.1	231.1	30	35
NAC-ss-Cys-162	283.1	162.0	30	22
NAC*-ss-Cys*-166	291.1	166.1	30	22
GSSG(+2)-130	307.1	130.1	30	17
GSSG*(+2)-130	310.1	130.1	30	17
GSSG(+2)-231	307.1	231.1	30	23
GSSG*(+2)-231	310.1	231.1	30	23
NAC-ss-162	325.1	162.0	30	20
NAC*-ss-166	333.1	166.0	30	20
Cys-NEM-126	247.1	126.0	47	35
Cys*-NEM-126	251.1	126.0	47	35
Cys-NEM-184	247.1	184.0	47	30
Cys*-NEM-186	251.1	186.0	47	30
GS-NEM-201	433.1	201.0	50	30
GS*-NEM-201	436.1	201.0	50	30
GS-NEM-304	433.1	304.0	50	25
GS*-NEM-307	436.1	307.0	50	25
Hcy-NEM-56	261.1	56.0	30	45
Hcy*-NEM-60	265.1	60.0	30	45
CG-NEM-201	304.1	201.0	30	20
CG-NEM-212	304.1	212.1	30	25
rEC-NEM-247	376.1	247.1	50	20
rEC-NEM-201	376.1	201.0	50	25
NAC-NEM-201	289.1	201.1	30	25
NAC*-NEM-204	293.1	204.1	30	25
NAC-NEM-230	289.1	230.0	30	20
NAC*-NEM-233	293.1	233.0	30	20
Caff*−140	198.1	140.1	55	28
Caff-138	195.1	138.1	55	28

### Plasma oxidation

Citrated plasma from a healthy donor was incubated with increasing concentrations of HOCl or H_2_O_2_ (Sigma-Aldrich) at 37 °C for 30 min. NEM was added to alkylate the thiols remaining at the end of the incubation period. Samples were aliquoted and stored at −80 °C until analysis.

### Reagents

The following reagents (with their sources indicated in parentheses) were used: acetonitrile and methanol (LC-MS grade, J.T. Baker), formic acid (EMD), dithiothreitol (Bio-Rad), N-ethylmaleimide (Pierce), sodium hypochlorite (Fisher Scientific), L-cysteine-^13^C_3_^15^N, N-acetyl cysteine-^13^C_3_^15^N, cystine-^13^C_6_^15^N_2_, homocysteine-3,3,4,4-d_4_, and caffeine-^13^C_3_ (Cambridge Isotope Laboratories). L-cysteine-glutathione disulfide (Cayman). All other reagents were obtained from Sigma-Aldrich.

**Table 2:** Composition and concentration of analytes in the QC standard (all thiols were blocked with NEM before adding to the mixture).

Name	Abbreviation	Conc (µM)
L-cysteine	Cys	8.0
Glutathione	GSH	2.2
N-acetyl cysteine	NAC	10.0
L-homocysteine	Hcy	3.0
L-cysteine-L-glycine	CG	3.0
γ-L-glutamyl-L-cysteine	γ-EC	1.1
Cystine	Cystine	15.4
Glutathione disulfide	GSSG	2.0
N-acetyl cysteine disulfide	NACss	8.0
L-cysteine-L-glycine disulfide	CGss	2.6
L-cysteine-glutathione disulfide	GSH-ss-Cys	6.0
L-cysteine-homocysteine disulfide	Hcy-ss-Cys	3.2
L-cysteine-N-acetyle cysteine disulfide	NAC-ss-cys	5.4
L-cysteine-CysGly disulfide	CG-ss-Cys	6.9
L-cysteine-γGluCys disulfide	γ-EC-ss-Cys	0.8
Caffeine	Caff	4.2

### Quality control (QC)

To monitor the variations among experiments, we included an aliquot of QC standard in each assay. The QC standard contained a mixture of non-isotopically labeled analytes with their concentrations listed in Table 2. The analytes in thiol form were blocked with NEM (1:4 molar ratio) at 37 °C for 30 min in 10 mM phosphate buffer, pH 6.0, before adding them to the mixture. Aliquots of the QC standard were stored at −80 °C until used. We also used the QC standard to quantify the concentrations of isotopically labeled analytes in internal standards.

### Internal standards

Internal standards contained a mixture of isotopically labeled analytes (indicated with *) with their concentrations listed in Table 3 (quantified relative to the QC standard). The analytes in thiol form were alkylated with NEM (1:4 molar ratio) at 37 °C for 30 min in 10 mM phosphate buffer, pH 6.0, before being added to the mixture. Disulfide-bonded analytes were generated as follows (final concentrations are listed): 1) for NAC*-ss-NAC* and NAC*-ss-Cys*, 2 mM NAC* was incubated with 1 mM cystine* in 20 mM ammonium bicarbonate buffer, pH 8.0, at 37 °C overnight, and the reaction mixture was then neutralized with phosphate buffer, pH 6.0, and blocked with NEM (1:4, molar ratio of thiols to NEM) to prevent further disulfide exchange; 2) GSSG* was generated by oxidizing 1 mM GSH* with 1 mM H_2_O_2_ (1:1 molar ratio) in 20 mM phosphate buffer, pH 7.0, for 2 hr, and the non-oxidized GSH* was blocked with 2 mM NEM. Concentrated internal standard (20×) was aliquoted, and stored at −80 °C. The 20× internal standard was diluted to 1× before use.

**Table 3:** Composition and concentrations of isotopically labeled analytes in the internal standard (all thiols were blocked with NEM before adding to the mixture).

Name	Abbreviation	1× int standard (µM)
L-cysteine-^13^C_3_, ^15^N	Cys*	9.0
Glutathione (glycine-^13^C_2_, ^15^N)	GSH*	1.7
N-acetyl cysteine-^13^C_3_, ^15^N	NAC*	6.1
Homocysteine-3,3,4,4-d_4_	Hcy*	3.0
Cystine-^13^C_6_, ^15^N_2_	Cystine*	16.0
Glutathione (glycine-^13^C_2_, ^15^N) disulfide	GSSG*	2.5
N-acetyl cysteine-^13^C_3_, ^15^N disulfide	NAC*ss	8.3
Cys*-NAC* disulfide	NAC*-ss-Cys*	20.4
Caffeine-^13^C_3_	Caff*	4.6

### Sample preparation

A 20 µl aliquot of NEM-treated frozen plasma was diluted with 100 µl of 5 mM phosphate buffer (1:6 dilution), pH 6.5. The diluted plasma was vortexed until completely thawed before mixing with 20 µl of 1× internal standard. The sample was used for different measurements indicated in Fig. 1a and quantification for total protein concentration.

To quantify individual small molecule thiols and disulfides, the sample (diluted plasma with NEM and internal standard, 40 µl) was incubated with 40 µl of 5 mM NEM in 5 mM phosphate buffer, pH 6.0, for 30 min at 37 °C, followed by protein precipitation with methanol (80% final v/v) at −20 °C for 1 hr. Proteins were removed by centrifugation at 20,000 g for 20 min at 4 °C, and supernatant was diluted 1 to 10 with 0.01% acetic acid for LC-MS/MS analysis.

To quantify total small molecule thiols and disulfides, the supernatant (40 µl) was subjected to a second protein precipitation with 120 µl cold methanol at −20 °C for 2 hr. The proteins were removed by centrifugation at 20,000 g for 20 min at 4 °C. The supernatant was vacuum dried and then reduced with 120 µl of 5 mM DTT in 20 mM phosphate buffer, pH 7.4, for 20 min at 65 °C^6,14^. After the reaction mixture was cooled to room temperature, the resulting thiols were blocked with 80 µl of 20 mM NEM in 5 mM phosphate buffer, pH 6.5, for 30 min at 37 °C. The sample was diluted 1 to 5 for LC-MS/MS analysis.

To quantify total thiols and disulfides, the samples (diluted plasma with NEM and internal standard, 20 µl) were reduced with 80 µl of 12 mM DTT in 20 mM phosphate buffer, pH 7.4, for 20 min at 65 °C and then alkylated with 40 µl of 60 mM NEM in 5 mM phosphate buffer, pH 6.5, for 30 min at 37 °C. Proteins in the samples were removed by methanol precipitation (80%, v/v) at −20 °C for 1 hr and centrifugation, and the supernatant was diluted 1 to 10 for LC-MS/MS analysis.

### Quantification of total protein concentration in plasma

The Bradford protein assay (Bio-Rad) was used to measure total protein concentration according to the manufacturer’s instructions using bovine serum albumin as the standard (Pierce^TM^ Bovine Serum Albumin Standard Ampules, catalog #: 23209, ThermoFisher Scientific).

### LC-MS/MS-MRM analysis

LC-MS/MS analysis was performed using a Waters ACQUITY I-Class ultra-performance liquid chromatograph coupled to a SCIEX QTRAP 6500 mass spectrometer with standard electrospray ionization source (ESI). Analytes were separated using a Waters HSS T3 column (2.1 mm × 100 mm × 1.8 µm) with column temperature at 40 °C. Mobile phase A was 0.1% formic acid in water and mobile phase B was 0.1% formic acid in acetonitrile. Samples (5 or 10 µl) were injected using an autosampler at 8 °C. Analytes were eluted at a flow rate of 0.3 ml/min with a gradient of increasing mobile phase B concentration, as follows: 0.2% for 1 min, 0.2% to 10% for 1 min, 10% to 25% for 4 min, and 25% to 90% for 2 min. The column was cleaned with 90% mobile phase B for 1.5 min and then equilibrated with 0.2% mobile phase B for 5 min. Analytes were detected in the mass spectrometer using MRM in positive mode under the following conditions: ion spray voltage: 5500 V, temperature: 550 °C, curtain gas: 40 psi, collision gas: high, ion source gas 1: 45 psi, ion source gas 2: 50 psi, entrance potential: 10 V, collision cell exit potential: 11 V. The detailed conditions for MRM transitions, optimized declustering potential (DP), and collision energy (CE) are listed in Table 1. All LC-MS/MS systems were controlled by Analyst® software version 1.6.2 (SCIEX) and MRM data were acquired using the same software.

### Data analysis

LC-MS/MS-MRM data was processed using MultiQuant 2.1 software (SCIEX). The peak area was used to quantify the concentration of analytes as follows:

A. If the isotopically labeled analyte was present in the internal standard, the concentration of the corresponding analyte in samples was calculated with equation (1):
  1 $$[a]=\frac{{(Peakarea)}_{a}}{{(Peakarea)}_{{a}^{\ast }}}\times [{a}^{\ast }]$$
  a* : the isotopically labeled analyte in the internal standard with known concentration (Table 3)
  a: the corresponding analyte
B. The concentration of protein-bound thiols was equal to the concentration of total thiols and disulfides minus the concentration of total small molecule thiols and disulfides, as shown in Fig. 1a.

### Statistical analysis

Unpaired t tests were used to compare the differences between healthy controls and patients with either SCD or sepsis. The correlation between protein-bound Cys and total Cys was evaluated with the Pearson correlation coefficient.

## Electronic supplementary material

Supplementary Information
