# Supplementary material for: Cysteine Disulfides (Cys-ss-X) as Sensitive Plasma Biomarkers of Oxidative Stress
Source: Sci Rep. 2019 Jan 14;9:115. doi: 10.1038/s41598-018-35566-2 (PMC6331564; doi:10.1038/s41598-018-35566-2)
Supplement: Supplementary file 1 — Supplementary Information [file 41598_2018_35566_MOESM1_ESM.pdf]

Supplementary Information

Cysteine Disulfides (Cys-ss-X) as Sensitive Plasma Biomarkers of Oxidative Stress

Xiaoyun Fu[1,2], Shelby A. Cate[1], Melissa Dominguez[1], Warren Osborn[1], Tahsin Özpolat[1], Barbara A. Konkle[1,2], Junmei Chen[1], José A. López[1,2]

[1] Bloodworks Research Institute, Seattle, USA

[2] Department of Medicine, University of Washington, Seattle, USA

Supplementary Table 1. Reproducibility, linear range, and limit of detection

Analytes	Reproducibility (RSD[&])		Linear range[#] (mol/L)	LOD[#] (mol/L)
	Intra-day (n=4)	Inter-day (n=8)		
Cys-NEM	2.8%	4.2%	$1.3 \times 10^{-5} – 4.0 \times 10^{-9}$	2.0×10^{-9}
GS-NEM	3.2%	3.0%	$2.0 \times 10^{-5} – 1.1 \times 10^{-9}$	5.0×10^{-10}
NAC-NEM	4.8%	4.5%	$1.2 \times 10^{-5} – 5.0 \times 10^{-9}$	2.5×10^{-9}
Hcy-NEM	2.2%	3.1%	$6.1 \times 10^{-6} – 3.0 \times 10^{-9}$	1.5×10^{-9}
CG-NEM	2.7%	5.4%	$1.1 \times 10^{-6} – 4.0 \times 10^{-9}$	2.0×10^{-9}
γEC-NEM	4.8%	5.9%	$6.0 \times 10^{-7} – 2.5 \times 10^{-9}$	1.2×10^{-9}
Cystine	2.0%	5.1%	$6.7 \times 10^{-6} – 3.8 \times 10^{-10}$	2.0×10^{-10}
GSSG	2.5%	6.5%	$7.7 \times 10^{-6} – 2.2 \times 10^{-9}$	1.0×10^{-9}
NACss	4.3%	4.8%	$1.2 \times 10^{-5} – 1.5 \times 10^{-9}$	7.5×10^{-10}
NAC-ss-Cys	1.8%	6.4%	$5.3 \times 10^{-7} – 2.7 \times 10^{-9}$	5.4×10^{-10}
Hcy-ss-Cys	0.9%	6.3%	$9.0 \times 10^{-7} – 4.5 \times 10^{-9}$	4.5×10^{-9}
CG-ss	3.8%	9.2%	$6.0 \times 10^{-7} –6.0 \times 10^{-9}$	3.0×10^{-9}
CG-ss-Cys	2.2%	6.8%	$7.0 \times 10^{-7} – 3.5 \times 10^{-9}$	2.0×10^{-9}
γEC-ss-Cys	4.0%	7.9%	$8.0 \times 10^{-7} – 1.6 \times 10^{-8}$	8.0×10^{-9}
GS-ss-Cys	4.4%	7.8%	$1.0 \times 10^{-6} – 5.0 \times 10^{-9}$	2.5×10^{-9}
Caffeine	2.9%	3.0%	$8.9 \times 10^{-6} – 3.6 \times 10^{-8}$	2.0×10^{-8}

[&]RSD: relative standard deviation
[#]Linear range and limit of detection (LOD) were tested with 5 µl of injection

Supplementary Table 2. Clinical characteristics of SCD patients

Patient ID	Ethnicity	Age	Sex	Genotypes	Hydroxyurea	Complications
1	African American	34	F	SS	No	Iron overload, pulmonary hypertension, acute chest syndrome, vasoocclusive crises, chronic renal failure, multiple strokes, deep vein thrombosis
2	African American	24	M	SS	Yes	Frequent vaso-occlusive crises, iron overload, multiple episodes of acute chest syndrome, pulmonary embolism (twice)
3	African American	22	F	SS	Yes	Frequent vaso-occlusive pain crises
4	African American	41	F	SS	Yes	Pulmonary hypertension, retinopathy, left shoulder and bilateral hip avascular necrosis, multiple deep vein thrombosis (chronically on anticoagulation)
5	African American	37	M	SS	Yes	Priapism, stroke, sickle cell hepatopathy, avascular necrosis of the femoral heads, iron overload
6	African American	61	F	SB^+	Yes	Bilateral hip avascular necrosis
7	African American	38	F	SS	Yes	Avascular necrosis both shoulders and knees, chronic severe pain
8	Hispanic	25	M	SB^0	Yes	Frequent vaso-occlusive pain crises, avascular necrosis of both hips and shoulders, transient ischemic attack
9	African American	28	M	SC	Yes	Acute chest sydrome (twice), priapism

Supplementary Table 3. Clinical characteristics of sepsis patients

Patient ID	Lived/Died	Age	Sex	ARDS[&]	APII[#]	APIII[#]
1	Lived	36	F	At risk	18	68
2	Died	73	M	At risk	24	79
3	Lived	48	F	Yes	27	84
4	Lived	57	F	At risk	20	52
5	Died	N/A	N/A	At risk	27	99

[&]**ARDS:** acute respiratory distress syndrome
[#]**APII/III:** Acute Physiology and Chronic Health Evaluation (APACHE) II/III scores, which correlate with disease severity. APII/III scores were assessed at the first evaluation after patients were admitted to the intensive care unit.

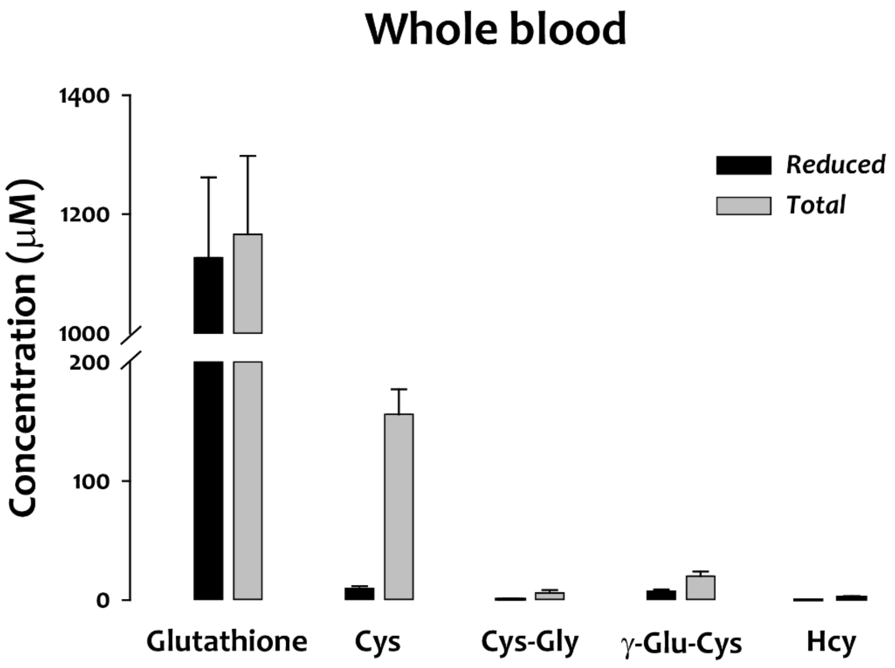

Supplementary Figure 1. Concentrations of reduced and total thiols in whole blood quantified by mass spectrometry. Whole blood from 11 heathy donors was collected in 3.2% sodium citrate then mixed with NEM (final concentration: 20 mM), aliquoted, snap-frozen in liquid nitrogen, and stored at -80°C until analysis. Reduced and total thiols in whole blood were analyzed using the same method as for plasma (see Methods) except that the whole blood samples were diluted 1 to10 with 5 mM phosphate buffer, pH 6.0, and the internal standard for whole blood quantification contained GSH* at 150 μM.
